# Quantitative CT reconstruction kernel harmonization for multi‐site lung cancer screening

**DOI:** 10.1002/acm2.70325

**Published:** 2025-11-07

**Authors:** David W. Jordan, Ryan E. Misseldine

**Affiliations:** ^1^ Department of Radiology University Hospitals Cleveland Medical Center Case Western Reserve University School of Medicine VA Northeast Ohio Healthcare System Cleveland Ohio USA; ^2^ Radiation Safety VA Northeast Ohio Healthcare System Cleveland Ohio USA

**Keywords:** kernel, lung, screening, texture

## Abstract

**Background:**

Published reference CT protocols for lung cancer screening are not optimized to produce uniform image appearance across different scanner manufacturers and models or to conform to quantitative imaging profiles for robust small lung nodule size and volume measurements, which are important in clinical management of screen‐detected nodules.

**Purpose:**

This study used widely available phantoms and software to identify lung cancer screening CT reconstructions that enable accurate and reproducible nodule size measurements and to match reconstructions across scanner manufacturers and models to provide a consistent image appearance to interpreting physicians.

**Methods:**

ACR CT accreditation phantom scans were used to measure the modulation transfer function (MTF) and noise power spectrum (NPS) for various reconstruction kernels for six CT scanner models from three manufacturers. A reference kernel was chosen, and other kernels were matched based on agreement between MTF and NPS for each candidate kernel. Kernels were validated for conformance to the Quantitative Imaging Biomarkers Alliance (QIBA) Small Lung Nodule profile using a commercial phantom and conformance testing software.

**Results:**

Medium sharp kernels with similar MTF and NPS curves and QIBA‐compliant imaging performance were identified for the six scanner models. One medium smooth kernel with iterative reconstruction and one common lung kernel did not conform to the QIBA profile.

**Conclusions:**

MTF and NPS comparisons to a validated reference CT protocol can be used to identify candidate scanner‐specific reconstructions appropriate for lung cancer screening that conform to the QIBA protocol, thus producing accurate and reproducible nodule size measurements, and that should provide uniform image appearance to the interpreting radiologist.

## 1 INTRODUCTION

Lung cancer screening (LCS) using computed tomography (CT) is growing in volume^1^ and importance^2^ for managing population health. Veterans Affairs (VA) has many patients who meet the criteria for routine LCS and cares for additional populations who may benefit from LCS due to exposure risks associated with military service. To manage these patients effectively, VA aims to implement a uniform LCS program across all its facilities nationally. This includes acquiring consistent CT imaging from different scanners in multiple facilities and standardizing the reporting of results and follow‐up. Standardization is of particular importance to VA because radiology exams scanned in each facility may be interpreted by radiologists affiliated with the individual VA facility, the VA National Teleradiology Program, or contracted individual radiologists or teleradiology practices.

A key image quality requirement in LCS is the detectability of suspicious nodules. Clinical trials of CT LCS indicate that nodule detectability for images acquired using typical CT parameters is sufficient to improve patient outcomes.^3^

The American College of Radiology (ACR) Lung‐RADS framework is used to standardize reporting in LCS. In the 2022 version of Lung‐RADS,^4^ the size of a nodule at detection and interval changes in nodule size are used to categorize risk and determine patient management. Accordingly, accurate and reproducible nodule size measurements are essential to proper patient management, and reliable measurements from the LCS CT study can save time and cost associated with additional follow‐up CT exams that would otherwise be needed as part of patient work‐up following a positive LCS CT study.

Technical requirements for accurate and reproducible lesion volume measurements were developed and published by the Quantitative Imaging Biomarkers Alliance (QIBA) in a quantitative imaging profile.^5^ This profile can be used to define an endpoint to optimize CT protocols, considering lesion size measurement performance as well as typical CT exam quality outcomes such as noise, blurring, contrast, detectability, and radiation exposure.

Due to growing interest in radiomic analysis and the use of artificial intelligence and machine learning methods to extract information from medical images, it is also prudent for large‐scale screening programs to standardize imaging data sets to facilitate such analyses in the future. Reconstruction kernels play an important role in radiomic characteristics of CT images, and the matching of CT kernels is an important precursor to quantitative image analysis.^6^, ^7^

Published scanner‐specific CT protocol recommendations are available for numerous CT scanner models^8^ to guide CT facilities in rapidly developing clinical protocols or for use as an external validation benchmark. These protocols appear to emphasize the nodule detection task. An inspection of the recommended reconstruction parameters reveals that these protocols are not harmonized among different scanner manufacturers and models, resulting in the delivery of exams with different characteristic appearances to interpreting radiologists in an organization with a mixed fleet of scanners. Specifically, the published protocols recommend different combinations of imaging planes, slice thicknesses, reconstruction kernel types, and default display states. Recent work has shown that protocols can be prospectively optimized using quantitative image quality features^9^ and translated successfully from a reference scanner to additional scanners in the field.^10^

This paper describes the process used by VA to harmonize CT reconstruction parameters for LCS to produce consistent LCS CT image sets for interpretation and digital analysis across the VA enterprise. We adopted the published American Association of Physicists in Medicine (AAPM) acquisition technique factors and developed harmonized reconstruction parameters using ACR guidelines and the QIBA Small Lung Nodule profile. The aims of the process were to deliver image sets specified by VA's national radiology leaders and to verify conformance to the QIBA Profile for reliable nodule size measurements. The process resulted in the creation of scanner‐specific LCS CT protocols suitable for dissemination throughout the VA National Radiology Program and a method and reference data set to extend this harmonization to additional scanners.

## 2 MATERIALS AND METHODS

We piloted the process described in this paper for several CT scanner models: GE Revolution CT and Revolution Apex (GE Healthcare, Waukesha, WI); Siemens Somatom Definition AS+, Somatom Definition Flash, and Somatom Drive (Siemens Healthineers, Forchheim, Germany); and Philips Spectral CT 7500 (Philips Healthcare, Haifa, Israel).

### 2.1 Parameter definition

We used the published reference LCS CT protocols^8^ as the baseline for developing harmonized protocols. We adopted the acquisition parameters, such as kV, detector configuration, pitch, rotation time, mA or automatic exposure control parameters, and target or expected CTDI_vol_, without modification.

We defined the reconstructed image series and parameter requirements, beginning with details in the ACR‐Society of Thoracic Radiology Practice Parameter for the Performance and Reporting of Lung Cancer Screening Thoracic Computed Tomography.^11^ We also incorporated recommendations listed in the technical specifications of the ACR Lung Cancer Screening Center Designation program website.^12^

To narrow the options for those reconstruction parameters that are not explicitly defined in the clinical and accreditation guidelines for LCS, we used the QIBA Profile for Small Lung Nodule Volume Assessment and Monitoring in Low Dose CT Screening.^5^ To ensure that the reconstruction results in a set of quantitative images conforming to the profile, we adopted the QIBA recommendation to use a reconstruction kernel “recommended to be a medium smooth to medium sharp kernel that provides the highest resolution available without edge enhancement.”

Where a parameter had different values recommended in multiple guidelines, we selected the most demanding or restrictive value; for example, where different sources recommended different reconstructed slice thicknesses, we chose the thinnest recommendation from among those options.

### 2.2 Kernel selection and characterization

For most CT scanners in the VA fleet, it was not possible to determine from documentation which of the available reconstruction kernels would best fit the QIBA recommendation. We selected the Chest kernel for GE scanners as the reference method since it was already in common use for clinical lung imaging and was shown by prior unpublished work in VA lung cancer screening programs to conform to the QIBA profile. We evaluated kernels from other scanners for similarity to the reference GE Chest images.

We defined matching modulation transfer function (MTF) and noise power spectrum (NPS) as closely as possible as the target for harmonization. To search for candidate reconstruction kernels on other scanners, we measured MTF and NPS for each kernel and compared the results to the measured MTF and NPS for the GE Chest kernel. We scanned the ACR CT Accreditation Phantom (Gammex Model 464, Sun Nuclear, Middleton, WI) using existing facility acquisition protocols for LCS and reconstructed contiguous axial slices (1 mm thickness) using each of the candidate kernels.

**FIGURE 1:**
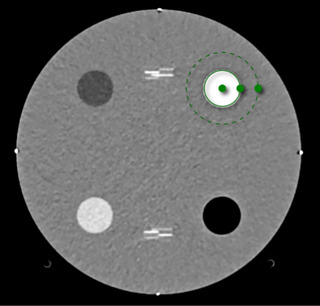
Example region of interest placement in the ACR phantom for measurement of the modulation transfer function.

We measured MTF for a stack of 20 contiguous slices using the Imquest web tool provided by Duke University (https://imquest.vm.duke.edu, Alpha Build, commit Jan 2, 2025). We measured MTF as the task transfer function (TTF) of the Teflon (bone‐like) phantom insert using the TTF tool ROIs placed as shown in Figure 1.^13^

**FIGURE 2:**
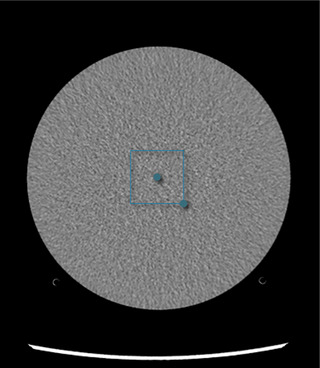
Example region of interest placement in the ACR phantom for measurement of the noise power spectrum.

We measured NPS^14^, ^15^, ^16^ in a stack of 20 contiguous slices from the uniform phantom region using the Imquest web tool. The ROI was placed as shown in Figure 2.

The Siemens B41 and B36 kernels were previously shown to produce similar noise textures to the GE Chest kernel,^16^ so we used these as initial candidates for Siemens CT scanners where equipped.

### 2.3 Validation

We scanned the Accumetra CTLX1 phantom (Accumetra, LLC, Clifton Park, NY) with the candidate acquisition and reconstruction protocols that produced MTF and NPS results most closely matching the reference results. We also reconstructed images using additional kernels and iterative reconstruction settings for illustrative purposes. We evaluated the resulting images for conformance to the QIBA profile in the categories of reconstructed image thickness, reconstructed image interval, resolution, edge enhancement, HU deviation, voxel noise, and spatial warping against the profile specifications. We uploaded the phantom images to the Accumetra ACQA CT Image Quality Assessment Software Platform (v 1.9) and downloaded the resulting QIBA Conformance Certification report to verify the results for each parameter and specification in the QIBA profile.

## 3 RESULTS

### 3.1 Kernel MTF measurements

**FIGURE 3:**
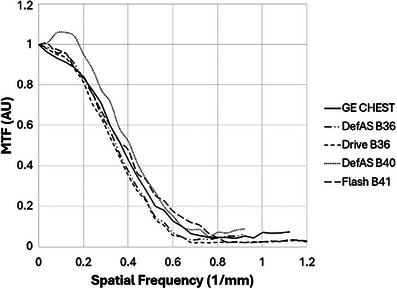
Modulation transfer function comparison of GE CHEST kernel with selected Siemens kernels.

**FIGURE 4:**
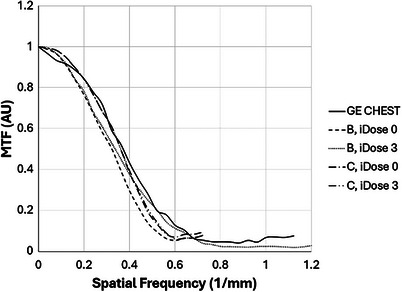
Modulation transfer function comparison of GE CHEST kernel with selected Philips kernels.

The measured MTF curves for the recommended kernels are plotted in Figures 3 and 4.

Based on the NPS agreement between the GE Chest kernel and the Siemens B36 and B41 kernels previously reported,^16^ we examined these as candidates for MTF matches. The MTF curves show good agreement among these three kernels; therefore, the Siemens B36 and B41 kernels would be expected to give similar spatial resolution performance to the GE Chest and to conform to those QIBA profile specifications that depend on spatial resolution. Both kernels had no edge enhancement, conforming to the QIBA profile. We also examined the B40 kernel for systems where the B41 is not available; this kernel showed some edge enhancement (less than 10%) and lesser agreement with the GE Chest MTF, confirming the B36 as the best match for systems that do not have B41 available.

Among the Philips kernels, the best match to the GE Chest was the C kernel with iDose level 3. The B kernel had lower spatial resolution than the GE Chest. Both kernels had no edge enhancement with or without iDose.

### 3.2 Kernel NPS measurements

**FIGURE 5:**
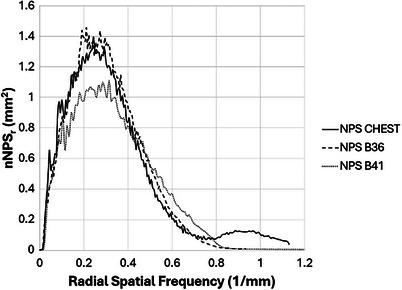
Noise power spectrum comparison of GE CHEST kernel with selected Siemens kernels.

**FIGURE 6:**
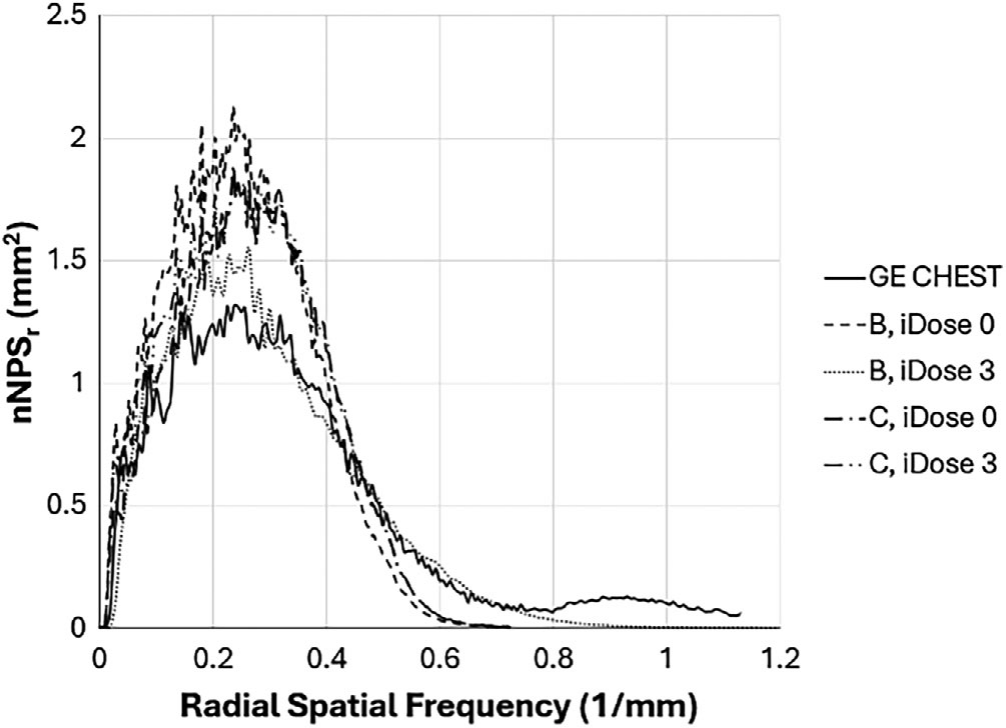
Noise power spectrum comparison of GE CHEST kernel with selected Philips kernels.

The measured NPS curves for the recommended kernels are plotted in Figures 5 and 6. The results confirm the agreement between the GE Chest kernel and the Siemens B41 and B36 kernels previously published.

The NPS for the Philips B kernel with iDose 3 shows excellent agreement with the GE Chest kernel. The B kernel with iDose 0 and the Philips C kernel with iDose 0 or 3 also exhibit good agreement, with apparent increases in noise amplitude and small shifts to higher spatial frequencies for the C kernel. All should have visual image texture appearances similar to images reconstructed with the GE Chest kernel, although some readers might observe differences with the C kernel images.

### 3.3 QIBA conformance validation

The results of the Accumetra QIBA conformance measurements are summarized in Table 1; “Pass” indicates that the CTLX1 phantom images met all the QIBA criteria checked by the Accumetra software.

**TABLE 1:** Results of Accumetra CTLX1 phantom validation tests for conformance to the QIBA Small Lung Nodule profile.

Scanner	Kernel	CTDIvol (mGy)	QIBA result
GE Revolution CT	Chest	1.8	Pass
Philips Spectral7500	B	2.5	Pass
Philips Spectral7500	B + iDose 3	2.5	Fail
Philips Spectral7500	C	2.5	Pass
Philips Spectral7500	C + iDose 3	2.5	Pass
Siemens Definition AS	Br36f	2.5	Pass
Siemens Definition AS	Br40f	2.5	Pass
Siemens Somatom Drive	Br36f	1.6	Pass
Siemens Somatom Drive	Br36f	2.6	Pass
Siemens Somatom Drive	Br36f / ADMIRE3	1.6	Pass
Siemens Somatom Drive	Br36f / ADMIRE3	2.6	Pass
Siemens Somatom Drive	Bl57f	1.6	Fail
Siemens Somatom Drive	Bl57f	2.6	Fail

The GE Chest, Siemens B36, and Philips B (iDose 0) and C (iDose 0 or 3) kernels all passed the Accumetra conformance test for the QIBA Small Lung Nodule profile when scanned using the reference CTDI_vol_ values listed in Table 1 and reconstructed using 1 mm axial slice thickness. This validation confirms that the tested protocols support accurate and reproducible nodule size measurements in clinical LCS. The Philips B, iDose 3 reconstruction was found to have insufficient 3‐dimensional spatial resolution on the Accumetra QIBA conformance test, consistent with its MTF, suggesting lower spatial resolution than the GE Chest, so the Philips B iDose 3 reconstruction was rejected as the preferred reconstruction despite its good NPS agreement with the GE Chest.

We also processed images reconstructed with the Siemens Bl57f kernel, which is commonly used in lung imaging, using the Accumetra QIBA conformance tool. These images failed the conformance check due to excessive edge enhancement and insufficient 3‐dimensional resolution aspect ratio, likely due to enhanced in‐plane resolution relative to the 1 mm image thickness. Therefore, these kernels are not appropriate for quantitative imaging in LCS.

### 3.4 Reconstruction parameters

The scanner‐specific protocol parameter recommendations we selected based on the ACR phantom measurements and best‐matched results are tabulated in Table 2. The reconstructions listed are for the primary axial image series used for radiologist interpretation as well as computerized analysis. The VA LCS CT protocol also includes additional reconstruction series for radiologist review, such as multi‐planar reformats; these are not included in Table 2.

**TABLE 2:** Recommended lung cancer screening protocols for six CT scanner models.

Scanner:	GE Revolution CT and Revolution Apex	Siemens Definition AS	Siemens Definition Flash	Siemens Somatom Drive	Philips Spectral CT 7500
Scan type:	Helical	Helical	Helical	Helical	Helical
Detector configuration:	128 × 0.625 mm	128 × 0.6 mm	128 × 0.6 mm	128 × 0.6 mm	64 × 0.625 mm
Pitch:	0.992	1.2	1.2	1.2	1
Rotation time (s):	0.35	0.5	0.5	0.5	0.4
KVp:	120	120	120	Sn100****	120
AEC settings:	AutomA, SmartmA, Noise Index 32 for first recon 5 × 3 mm (Chest)	CAREDose4D, 20 mAs reference; CAREkV, reference 120, optimize @ Chest	CAREDose4D, 20 mAs reference; CAREkV, reference 120, optimize @ Chest	CAREDose4D, 81 mAs reference, CAREkV	DoseRight DRI = 5
Scan options:	Helical plus	None	None	None	None
CTDIvol target or estimate (mGy):	2.3	1.3	1.3	0.6	1.7
Reconstruction plane:	Axial	Axial	Axial	Axial	Axial
Reconstruction kernel:	CHEST	Br36f	Br41f	Br36f	C
Reconstruction options:	None (ASiR‐V off)	None (SAFIRE off; ADMIRE off)	None (SAFIRE off; ADMIRE off)	None (SAFIRE off; ADMIRE off)	None (iDose 0)
Slice Thickness (mm):	1	1	1	1	1
Slice Interval (mm):	1	1	1	1	1
Display default (WW/WL):	1500 / −600	1500 / −600	1500 / −600	1500 / −600	1500 / −600

## 4 DISCUSSION

**FIGURE 7:**
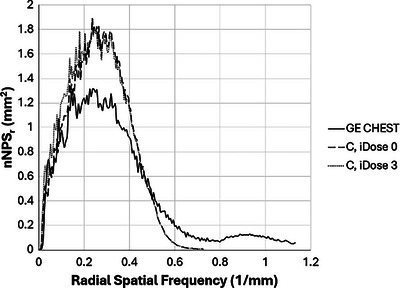
Noise power spectrum comparison showing increased low‐frequency content in Philips C kernel images using iDose 3.

The NPS results for the Philips C kernel with and without iDose 3 were very similar, but a closer inspection (Figure 7) reveals that the iDose 3 images contain elevated noise amplitudes at lower spatial frequencies. This is likely to introduce visual differences in images that may be distracting to readers. The C kernel without iDose (iDose level 0) was chosen as the best match from among the candidates that passed the QIBA conformance check.

There has been progress in lung cancer screening CT protocol optimization since the inception of screening programs,^17^ but optimization remains challenging due to advances in scanner technology.^18^, ^19^ Another challenge in optimization is defining the goal; numerous studies rely on qualitative reader studies or non‐generalizable quantitative methods, such as noise magnitude measurements, to evaluate options. The QIBA Small Lung Nodule profile provides quantitative image metrics that are important in the specific setting of LCS and that directly address the clinical tasks at hand, namely, detection of nodules and measurements of nodule size and volume. Our results show that typical thoracic CT reconstruction kernels are not adequate for LCS, emphasizing the point that LCS is a distinct diagnostic task from routine thoracic CT, so for best performance, LCS CT protocols need to be optimized to this specific task rather than adapted from routine thoracic CT protocols.

The approach described in this study could be extended to other scanner models and manufacturers by making MTF and NPS measurements for a variety of reconstruction kernels to identify suitable candidates and then validating the candidates using the QIBA Small Lung Nodule profile assessments, resulting in imaging protocols that are robust for quantitative nodule measurements, consistent in visual appearance for interpreting radiologists, and harmonized for radiomic and other computerized analysis.

A limitation of this study is that we did not evaluate quantitative harmonization of the image performance. While our results and method should provide similar visual texture for interpreting radiologists and conform to the QIBA profile for quantitative measurements, we did not assess measurement bias that may exist between our harmonized protocol pairs. For example, the Philips B, iDose 0 result has lower MTF values than the GE Chest. Both conform to the QIBA profile, suggesting that both have sufficient spatial resolution (and hence sufficiently limited partial volume effect) for reliable nodule size or volume measurements. However, we did not quantitatively assess whether paired nodule measurements from the two techniques agree, and it is reasonable to expect the MTF difference to introduce some bias in size measurements due to the partial volume effect. It is also unclear whether such differences could have a meaningful impact on the results of radiomic, artificial intelligence, or machine learning analysis or processing; in any case, it is likely that the increased harmonization achieved by our approach would reduce bias and variation in large data sets, compared to the case of a much greater variety of reconstructions in use.

Another limitation is that our results may not be suitable for the harmonization of images for radiomic analysis. We did not evaluate radiomic features in this study as other investigators have done.^7^ While we expect that matched MTF and NPS would reduce scanner‐specific or kernel‐specific differences in radiomic features, we did not analyze this outcome.

A further potential limitation is that the evaluation of patient images using the selected reconstructions was outside the scope of this study. Facilities employing this approach should consider conducting follow‐up reviews to ensure images appear uniform to radiologists and to evaluate clinical images using quantitative measures of image characteristics. Such evaluations would also be suitable subjects for future research in this area.

## 5 CONCLUSION

Since multiple CT reconstruction settings can enable robust quantitative nodule measurements in LCS, choosing kernels with matching MTF and NPS is a practical approach to benchmarking and harmonizing images for consistent radiologist presentation and interpretation, including among different scanner manufacturers and models. These measurements can be performed using the ACR CT accreditation phantom and free Web‐based software that should be available to most facilities. The proposed method was operationalized by defining LCS protocols for six CT scanner models from three manufacturers.

## AUTHOR CONTRIBUTIONS

*Contributed to the design and performance of the experiments, to the analysis of the results, and to the writing of the manuscript*: David W. Jordan and Ryan E. Misseldine.

## CONFLICT OF INTEREST STATEMENT

The authors declare no conflicts of interest.
